# CRLF1 Drives Prostate Cancer Progression via COMP-Mediated Activation of the FAK/PI3K/AKT Signaling Pathway

**DOI:** 10.3390/cancers18091395

**Published:** 2026-04-28

**Authors:** Zhongze Li, Jinrun Wang, Lizhe Xu, Jinzhuo Ning, Fan Cheng

**Affiliations:** Department of Urology, Renmin Hospital of Wuhan University, Wuhan 430060, China; 2024283020145@whu.edu.cn (Z.L.); 2019305232004@whu.edu.cn (J.W.); xlz460@whu.edu.cn (L.X.); njz120511@whu.edu.cn (J.N.)

**Keywords:** PCa, CRLF1, COMP, tumor progression, biomarker

## Abstract

**Background:** Cytokine-like receptor family 1 (CRLF1) has been implicated in tumor progression, yet its prognostic function and mechanistic actions in prostate cancer (PCa) remain elusive. **Objective:** This investigation sought to clarify the functional role, molecular mechanisms, and clinical relevance of CRLF1 in the progression of PCa. **Methods:** We conducted extensive bioinformatics analyses utilizing the protein interaction networks and the TCGA-PRAD dataset. CRLF1 and cartilage oligomeric matrix protein (COMP) expression were validated in clinical samples by qRT-PCR and Western blot (WB). Functional assessments, including Transwell invasion, flow cytometry, CCK-8, and wound healing, were conducted in vitro. An in vivo xenograft tumor model was used for further validation. Mechanistic investigations involved genetic perturbation (overexpression and inhibition) of CRLF1 and COMP. **Results:** Compared to benign tissues, the levels of CRLF1 and COMP were markedly elevated in PCa tissues. Bioinformatics assessments illustrated a robust positive relationship between CRLF1 and COMP, suggesting COMP may function as a downstream mediator. In vitro and in vivo investigations illustrated that silencing CRLF1 significantly suppressed PCa cell growth, invasion, and tumor progression, while enhancing apoptosis. Importantly, suppressing COMP counteracted the cancer-promoting effects triggered by CRLF1 overexpression. At the mechanistic level, CRLF1 facilitates tumor progression by modulating COMP to activate the FAK/PI3K/AKT signaling cascade. **Conclusions:** Our outcomes demonstrate that CRLF1 promotes PCa progression by targeting COMP to stimulate the FAK/PI3K/AKT signaling axis. This newly identified CRLF1/COMP/FAK/PI3K/AKT pathway underscores CRLF1 as a potential biomarker and therapeutic target for PCa.

## Simple Summary

Prostate cancer is one of the most common malignancies in men, yet the molecular mechanisms driving its progression remain incompletely understood. In this study, we identified cytokine-like receptor factor 1 (CRLF1) as a key regulator of prostate cancer progression. We found that CRLF1 promotes tumor cell proliferation, migration, and invasion while inhibiting apoptosis. Mechanistically, CRLF1 exerts its oncogenic effects by upregulating cartilage oligomeric matrix protein (COMP), which subsequently activates the FAK/PI3K/AKT signaling pathway. Importantly, the suppression of COMP attenuated the tumor-promoting effects of CRLF1, indicating that COMP acts as a critical downstream mediator. These findings support the relevance of a CRLF1/COMP-associated mechanism linked to FAK/PI3K/AKT signaling in prostate cancer and suggest that CRLF1 may serve as a potential biomarker and therapeutic target.

## 1. Introduction

Prostate cancer (PCa) is a predominant malignancy in men and is considered the 2nd major cause of cancer-related death in numerous countries [1]. Data from the Global Agency for Cancer Research indicate that the incidence of PCa has risen markedly over recent decades, particularly in North America and Europe [2,3]. This upward trend is linked to several contributing factors, such as population aging, improvements in screening methodologies, and heightened public awareness regarding PCa. Early identification of PCa predominantly depends on prostate-specific antigen (PSA) testing, and the widespread adoption of this assay has substantially elevated the detection rate of early-stage disease [4,5,6].

In terms of diagnosis and treatment, PCa management strategies have undergone significant evolution. Traditional treatment methods include surgery, radiotherapy, and hormone therapy, but with the advent of targeted therapy and immunotherapy, PCa treatment options have become increasingly rich [7,8]. Targeted therapy, especially targeting androgen receptors, has become an important strategy for the treatment of metastatic PCa [9,10]. In recent years, second-generation androgen receptor antagonists (e.g., enzalutamide and abiraterone) have shown significant efficacy in castration-resistant PCa, significantly prolonging survival [11,12]. Chemotherapy options, such as docetaxel, are commonly used to treat advanced or metastatic PCa, especially after hormone therapy has failed. Studies have shown that chemotherapy improves survival, especially in high-risk patients [13,14]. However, side effects and drug resistance remain challenges in clinical treatment, and new therapeutic strategies and drug combinations are urgently needed to improve efficacy [15,16]. Consequently, elucidating the molecular mechanisms underlying PCa progression and developing more effective targeted therapies are critically important.

Cytokine-like receptor family 1 (CRLF1) is a cytokine-like receptor-like protein. It was initially discovered due to its mutations in rare genetic diseases, and subsequently exhibited important regulatory functions in various tumors and tissue pathological states [17,18]. CRLF1 has been implicated in influencing disease progression across multiple pathological processes. With the advancement of molecular biology and genomics technologies, the biological functions of CRLF1 have been progressively elucidated. Notably, its involvement in tumor cell signaling, the regulation of the tumor microenvironment, tissue fibrosis, and regenerative repair has garnered significant attention. The tumors’ malignant progression and chemotherapy resistance are the main challenges in current cancer treatment, and CRLF1, which regulates cell proliferation, migration, apoptosis, and inflammation through multiple signaling pathways, provides new targets for tumor treatment [17,18,19]. According to published literature, CRLF1 has a vital oncogenic function in various tumors. In ovarian cancer, CRLF1 acts as a novel component of the mTORC2 complex, elevating the robustness of the interaction between AKT and SIN1 to activate the AKT signaling pathway. This activation suppresses pyroptosis and contributes to the development of chemoresistance [20]. In papillary thyroid cancer, elevated CRLF1 expression correlates with unfavorable prognosis and facilitates tumor aggressiveness and epithelial–mesenchymal transition (EMT) by the stimulation of the MAPK/ERK and PI3K/AKT pathways. Additionally, its metastatic role is further demonstrated to rely on its interaction with the MYH9 protein and the activation of the ERK/ETV4/MMP1 axis [21,22]. In colorectal cancer, CRLF1 serves as a direct target for oncogenic miR-3065-3p, which inherently possesses a function to inhibit tumor stem cell characteristics [23]. These findings collectively suggest that CRLF1 facilitates tumor growth, metastasis, stem cell properties, and therapeutic resistance by regulating multiple central signaling pathways. Nevertheless, the precise predictive significance and potential pathway mechanisms of CRLF1 in PCa continue to be fully clarified.

To examine this hypothesis, we conducted a comprehensive investigation combining clinical bioinformatics, molecular biology, and functional preclinical models. We first validated the clinical overexpression and correlation of CRLF1 in PCa. Through a series of gain-of-function and loss-of-function investigations conducted in vitro and in vivo, we systematically explained the oncogenic roles of CRLF1 and identified cartilage oligomeric matrix protein (COMP) as a functional mediator associated with CRLF1 signaling. These outcomes illustrated a previously undetected and druggable signaling cascade, establishing CRLF1 as a prospective indicator and a viable therapeutic target for advanced PCa.

## 2. Experimental Procedures

### 2.1. Database

Publicly available RNA-sequencing expression data and the corresponding clinicopathological and survival information for prostate adenocarcinoma were obtained from The Cancer Genome Atlas Prostate Adenocarcinoma cohort (TCGA-PRAD) through the Genomic Data Commons (GDC) Data Portal (Project ID: TCGA-PRAD; dbGaP study accession: phs000178; cohort page: https://portal.gdc.cancer.gov/projects/TCGA-PRAD; accessed on 21 July 2025). The Gene Expression Omnibus (GEO) dataset GSE46602 was used for external validation. Protein–protein interaction analysis was performed using the STRING database (Search Tool for the Retrieval of Interacting Genes/Proteins), version 12.0, for Homo sapiens (NCBI taxonomy ID: 9606; https://string-db.org/; accessed on 29 December 2025).

### 2.2. Bioinformatics

All bioinformatics analyses were performed using R software version 4.2.1. DESeq2 was utilized to determine differentially expressed genes (DEGs), with gene levels quantified as fragments per kilobase of transcript per million mapped reads (FPKM). Cox proportional hazards regression and survival analyses were performed using the survival package in R. Visualization of the results was performed using the survminer package and ggplot2 package version 3.4.4. Correlation analyses were carried out using Spearman’s rank correlation coefficient via the cor.test function in R. Gene Ontology (GO), Gene Set Enrichment Analysis (GSEA), and Kyoto Encyclopedia of Genes and Genomes (KEGG) enrichment analyses were performed using the clusterProfiler package version 4.4.4.

### 2.3. Clinical Samples

A total of 17 clinical tissue specimens, including prostate cancer tissues and adjacent non-tumorous tissues, were collected from patients who underwent radical prostatectomy at Renmin Hospital of Wuhan University from 2021 to 2026 for qRT-PCR validation. Three representative fresh tissue specimens were used for Western blot analysis. The study protocol was approved by the Institutional Review Board (IRB) of Renmin Hospital of Wuhan University and conducted in accordance with the Declaration of Helsinki. The requirement for informed consent was waived by the IRB due to the retrospective use of anonymized clinical specimens.

### 2.4. Cell Culture and Transfection

The source of the human PCa cell lines (22RV1, PC-3, and DU145), as well as the normal prostate epithelial cell line (RWPE-1), was the American Type Culture Collection (ATCC). We seeded 22RV1 cells in RPMI-1640 medium, PC-3 cells in Ham’s F-12K medium, DU145 cells in Minimum Essential Medium (MEM) with non-essential amino acids (NEAA), and RWPE-1 cells in Keratinocyte-SFM (K-SFM). The incubation of all cell lines was conducted at 37 °C in a humidified environment with 5% CO2. Procell Biotech Co., Ltd. (Procell, Wuhan, China). was the source of culture media and supplements. Basal media were enriched with 10% fetal bovine serum and 1% penicillin/streptomycin (Procell). Lentiviral vectors for the upregulation and knockdown of CRLF1 and COMP were procured from GeneChem. DU145 cells were transduced with lentivirus using HiTransGP infection enhancer at a multiplicity of infection (MOI) of 20. Post-transduction, cells were chosen with puromycin (4 µg/mL) for one week, and transfection efficiency was validated by qRT-PCR and WB.

### 2.5. Western Blot (WB) Assay

Protein extraction from both cultured cell lines and tissue specimens was performed utilizing the BCA Protein Assay Kit (Beyotime, Shanghai, China). Protein separation was achieved through electrophoresis on a 10% SDS-polyacrylamide gel (Solarbio, Beijing, China), after which we transferred the proteins onto a polyvinylidene fluoride (PVDF) membrane (Beyotime). For the blockage of non-specific binding positions, a 1-h incubation of the membrane was conducted at room temperature in phosphate-buffered saline (PBS) with 5% skim milk. The membrane was then subjected to three washes, each lasting 5 min, utilizing Tris-buffered saline with Tween-20 (TBST). Afterward, an incubation of the membrane was conducted overnight at 4 °C with these primary antibodies: CRLF1 (ab211438), p-AKT (ab38449), COMP (ab231977), FAK (ab40794), p-PI3K (ab182651), AKT (ab8933), p-FAK (ab81298), PI3K (ab302958), and GAPDH (ab8245) (All Abcam, 1:2000; except GAPDH 1:5000, Cambridge, UK). Following another three TBST washes, a 1-h membrane incubation was conducted at room temperature with horseradish peroxidase-conjugated secondary antibodies (ab6721 or ab205719, Abcam, dilution 1:5000). The visualization of protein bands was conducted via enhanced chemiluminescence (ECL) detection methods. The uncropped original Western blot images with molecular weight markers are provided in Supplementary Figures S2 and S3.

### 2.6. RNA Isolation and qRT-PCR

As per the manufacturer’s guidelines, total RNA extraction from human cells and tissues was conducted via TRIzol reagent (Solarbio). First-strand cDNA synthesis was conducted with a reverse transcription system kit (Vazyme, Nanjing, China). We conducted quantitative PCR via ChamQ Universal SYBR qPCR Master Mix (Vazyme) on a CFX96 real-time PCR detection system (Bio-Rad, Hercules, CA, USA). The thermal cycling parameters were applied as specified. The primer sequences used for qRT-PCR are listed in Table 1.

### 2.7. Cell Proliferation Assay

The Cell Counting Kit-8 (CCK-8; Beyotime) was conducted to assess cell viability and growth. PCa cells were plated in plates with 96 wells (2000 cells/well). Cell proliferation was evaluated at 0, 24, 48, and 72 h. Following incubation, CCK-8 reagent (10 µL/well) was applied, and an incubation of the plates was conducted for 2 h per the manufacturer’s guidelines. Utilizing a microplate reader, absorbance was observed at 450 nm. Cell viability was normalized to the corresponding control group and expressed as percentage viability.

### 2.8. Cell Apoptosis Assay

Cells were rinsed and detached using trypsin without EDTA. Following spinning and rinsing with ice-cold PBS, the cells were gently resuspended in chilled 1× binding buffer. We estimated apoptosis via an Annexin V-FITC/PI Apoptosis Detection Kit (Elabscience, Wuhan, China) as per the manufacturer’s guidelines. Flow cytometry was employed to estimate the samples and quantify apoptotic cells.

### 2.9. Wound Healing Assay

Cells were cultivated in six-well plates with suitable pore sizes and incubated overnight until they reached full confluence. A consistent linear scratch was introduced via a sterile 100 μL pipette tip. The wound’s width was recorded right after the scratch was made and then again after incubating for 24 h. The areas devoid of cells were assessed by analyzing digital images of the wound site in comparison to the surrounding confluent regions.

### 2.10. Transwell Assay

Cell invasion was estimated via Transwell inserts (8.0 μm pore size; Millipore, Burlington, MA, USA) pre-coated with Matrigel (BD Biosciences, San Jose, CA, USA). Briefly, 100 μL of serum-free MEM with NEAA and transfected PCa cells was introduced into the top chamber, while the bottom chamber encompassed 500 μL of MEM with NEAA and 10% fetal bovine serum as a chemoattractant. After incubating for 48 h, invaded cells underwent fixing with 4% paraformaldehyde, staining with crystal violet, and counting via a light microscope.

### 2.11. Immunohistochemistry (IHC) Staining

First, tumor and neighboring tissue samples underwent fixing in 4% paraformaldehyde, then paraffin-embedding, slicing, and staining. The immunohistochemically stained sections are processed using the SP kit (Streptavidin-Peroxidase detection system) from ZSGB-BIO (ZSGB-BIO, Beijing, China), following the manufacturer’s guidelines. Then, we incubated the slices overnight with primary antibodies against CRLF1 (ab211438, Abcam, 1:100) and Ki67 (ab16667, Abcam, 1:200) at 4 °C, and then incubation with HRP-conjugated secondary antibody was conducted for 30 min at 37 °C. Positive signals are observed with DAB chromogen, and images are acquired under a microscope. Staining was assessed using two complementary methods: the proportion of positive cells and the immunoreactivity score. Cells with distinct brown cytoplasmic or nuclear staining were considered positive, and their proportion relative to total cells was calculated. The immunoreactivity score was calculated by semi-quantitatively assessing staining intensity (0 = negative, 1 = weak, 2 = moderate, 3 = strong) and applying: Immunoreactivity score = (percentage of weak × 1) + (percentage of moderate × 2) + (percentage of strong × 3), yielding a range of 0–300. All scoring was performed independently by two experienced pathologists who were blinded to the clinical data.

### 2.12. In Vivo Experiment

The Animal Care and Use Ethics Committee of Renmin Hospital, Wuhan University gave its approval to all animal trials, which were conducted as per institutional protocols. BALB/c nude mice (male, four weeks old) were acquired from Wuhan WQJX Biotechnology Co., Ltd (WQJX Biotechnology Co., Ltd., Wuhan, China). and acclimated for one week before the investigation. Mice were classified into two experimental groups in a random manner (*n* = 3/group). Heterotopic tumor models were created by subcutaneous injection of 150 μL PBS containing stably transfected DU145 cells into the ventrolateral flank of each mouse. Tumor dimensions (length a and width b) were assessed weekly with calipers, and tumor volume was measured as $ab^{2}/2$. At week four, mice were euthanized, tumors excised, and weights recorded.

### 2.13. Statistical Analysis

GraphPad Prism (v 10.4) was employed to conduct statistical analyses. Results from three independent trials are represented as mean ± standard deviation. We assessed two-group variations via two-tailed unpaired Student’s *t*-tests. For multiple-group comparisons under a single factor, one-way analysis of variance (ANOVA) and then Tukey’s post hoc test were utilized. In trials with two factors (such as overexpression and knockdown conditions), we utilized two-way ANOVA with Tukey’s post hoc test. For time-series measurements (e.g., CCK-8 assays and tumor volume tracking), repeated measures ANOVA or one-way ANOVA, depending on the area under the curve analysis, was utilized to estimate differences. Significance was set as *p* < 0.05.

## 3. Results

**Figure 1:**
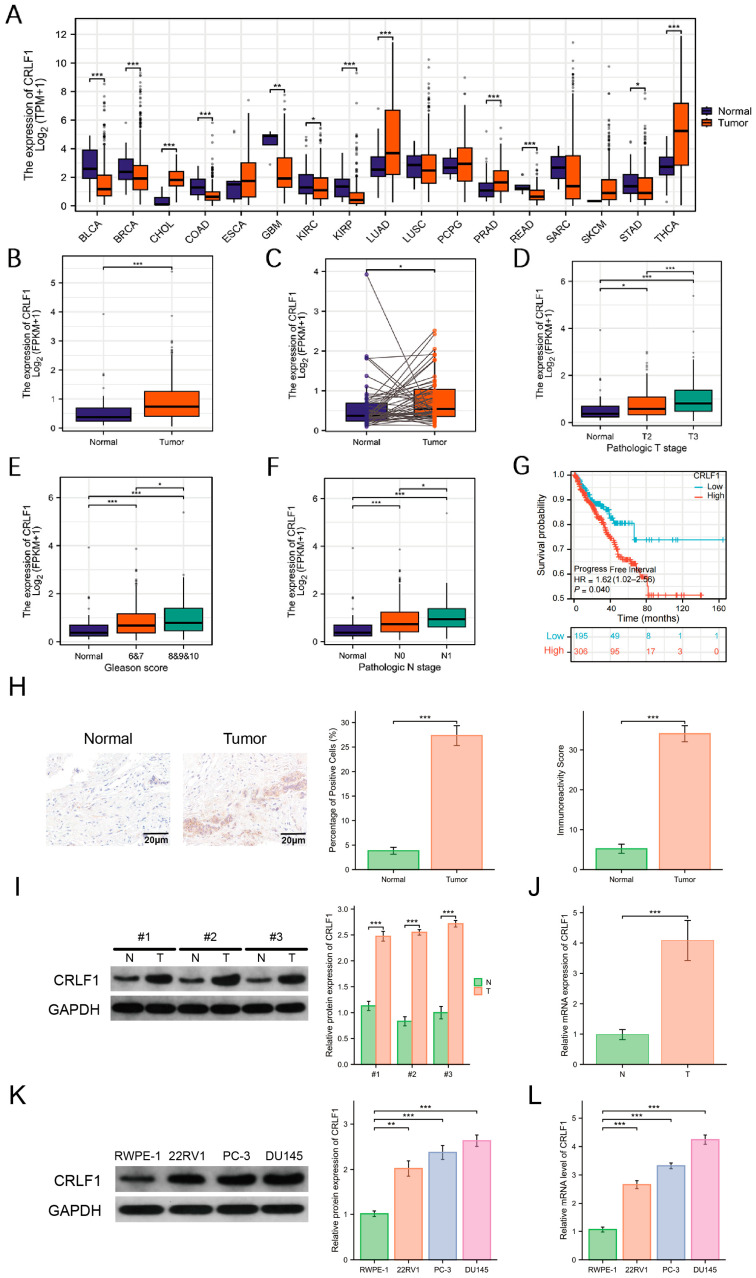
CRLF1 is upregulated in PCa and relates to advanced clinical stage and poor prognosis. (**A**) CRLF1 expression across multiple cancers using TCGA data. (**B**,**C**) CRLF1 mRNA levels in PCa versus normal tissues from TCGA-PRAD. In panel (**C**), the lines connect paired normal and tumor samples from the same patient. (**D**–**F**) Association of CRLF1 expression with T stage, Gleason score, and N stage in TCGA-PRAD. (**G**) Progression-free interval (PFI) survival curves stratified by CRLF1 expression. (**H**) Immunohistochemical staining showed that CRLF1 positive rate and immunoreactivity score in tumor tissues were significantly increased compared to healthy tissues (*n* = 3). (**I**,**J**) WB (*n* = 3) and qRT-PCR (*n* = 17) of CRLF1 in PCa tissues and neighboring healthy tissues. (**K**,**L**) WB and qRT-PCR of CRLF1 level in PCa cell lines (22RV1, PC-3, DU145) versus non-cancerous RWPE-1 cells (*n* = 3). Data are mean ± SD. * *p* <0.05; ** *p* < 0.01; *** *p* <0.001. The corresponding uncropped original Western blot images with molecular weight markers are provided in Supplementary Figure S2A–D.

CRLF1 expression is noticeably heightened in PCa and shows a significant correlation with advanced clinical stages and poorer prognosis. TCGA data analysis revealed that CRLF1 was dysregulated across multiple tumor types rather than uniformly elevated, with increased expression in several cancers and decreased expression in some others (Figure 1A). Both in unpaired and paired sample datasets, CRLF1 exhibited significantly higher expression in PCa (Figure 1B,C). We collected three tumor and healthy tissues, and immunohistochemical staining confirmed that CRLF1 was significantly higher in tumor tissue in terms of cellular positivity and immunoreactivity scores (Figure 1H). WB analysis of three representative paired clinical samples and qRT-PCR analysis of 17 clinical tissue specimens, including cancer and adjacent non-tumorous tissues, further confirmed that CRLF1 expression was markedly elevated in PCa tissues compared to healthy tissues (Figure 1I,J). Furthermore, Kaplan–Meier survival curves demonstrated a significant relationship between elevated CRLF1 levels and progression-free survival (PFI) in individuals diagnosed with PCa (Figure 1G), with the dataset used in this analysis being TCGA-PRAD. Further investigation of the TCGA database illustrated significant correlations between CRLF1 level and numerous clinical parameters, such as T stage, Gleason score, and N stage (Figure 1D–F). To further validate the TCGA-derived findings, we performed external analyses in independent public datasets, which confirmed CRLF1 dysregulation in prostate cancer and further evaluated its correlations with COMP (Supplementary Figure S1).

### 3.1. CRLF1 Promotes Growth and Inhibits Apoptosis of PCa Cells

**Figure 2:**
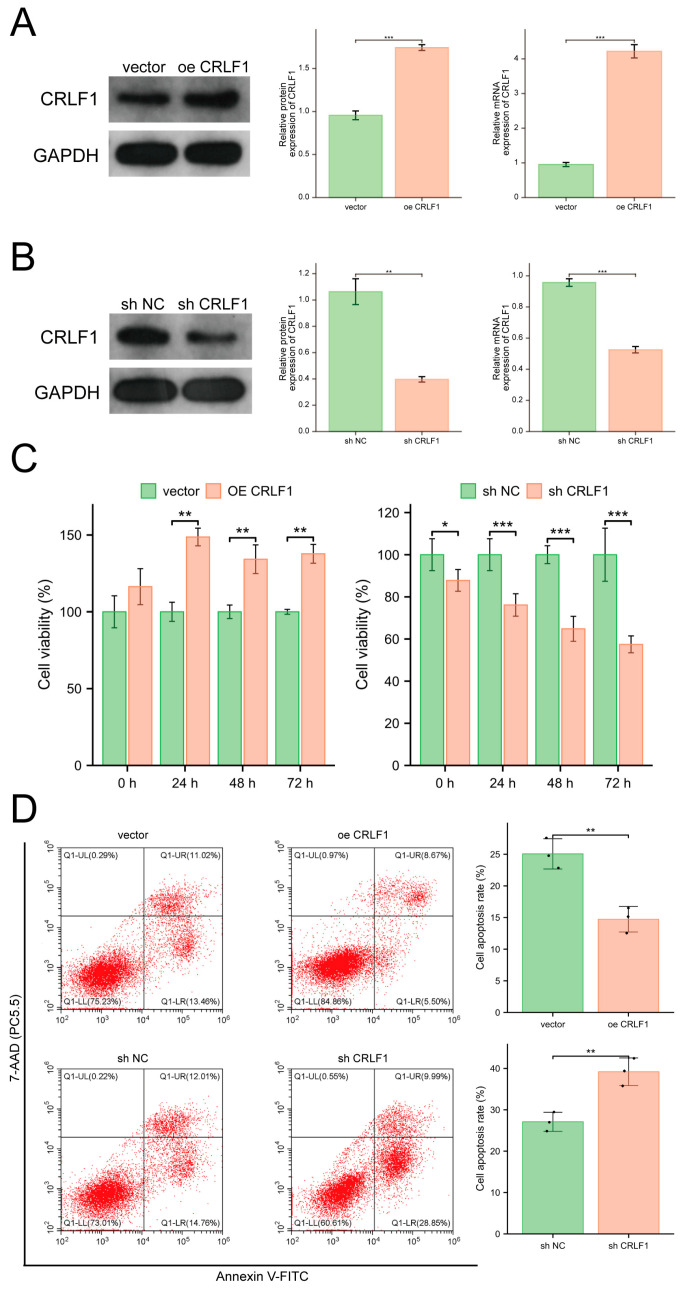
CRLF1 promotes growth and inhibits apoptosis in PCa cells. (**A**,**B**) WB and qRT-PCR confirmed CRLF1 overexpression and knockdown in DU145 cells. (**C**) CCK-8 assay measured cell viability at 0, 24, 48, and 72 h after CRLF1 modulation (*n* = 5). Values were normalized to the corresponding control group at each time point and expressed as percentage viability. (**D**) Flow cytometry analysis of apoptosis in DU145 cells following CRLF1 overexpression or knockdown (*n* = 3). Data are mean ± SD. * *p* <0.05; ** *p* < 0.01; *** *p* <0.001. The corresponding uncropped original Western blot images with molecular weight markers are provided in Supplementary Figure S2E–H.

WB and qRT-PCR analyses of CRLF1 expression (Figure 1K,L) revealed a marked increase in CRLF1 levels in PCa cell lines 22RV1, PC-3, and DU145 relative to the benign prostate epithelial cell line (RWPE-1). Among these, DU145 cells depicted the highest CRLF1 expression and were therefore chosen for subsequent experimental investigations. Although CRLF1 expression was also elevated in 22RV1 and PC-3 cells, 22RV1 harbors an AR-variant background, whereas PC-3 is PTEN-deficient, both of which may introduce additional complexity in interpreting CRLF1-associated signaling. To explore the function of CRLF1 in PCa, stable DU145 cell lines with either enhanced or suppressed CRLF1 expression were established. The successful modulation of CRLF1 expression was validated by WB and qRT-PCR analyses (Figure 2A,B). CCK-8 assays performed at 0, 24, 48, and 72 h illustrated that CRLF1 suppression significantly impaired PCa cell growth, while CRLF1 overexpression substantially improved it (Figure 2C). Furthermore, flow cytometric analysis demonstrated that CRLF1-overexpressing PCa cells depicted a significantly reduced apoptosis rate compared to the negative control group. Conversely, CRLF1-deficient cells showed a pronounced elevation in apoptosis (Figure 2D).

### 3.2. CRLF1 Facilitates the Migration and Invasion of PCa Cells

**Figure 3:**
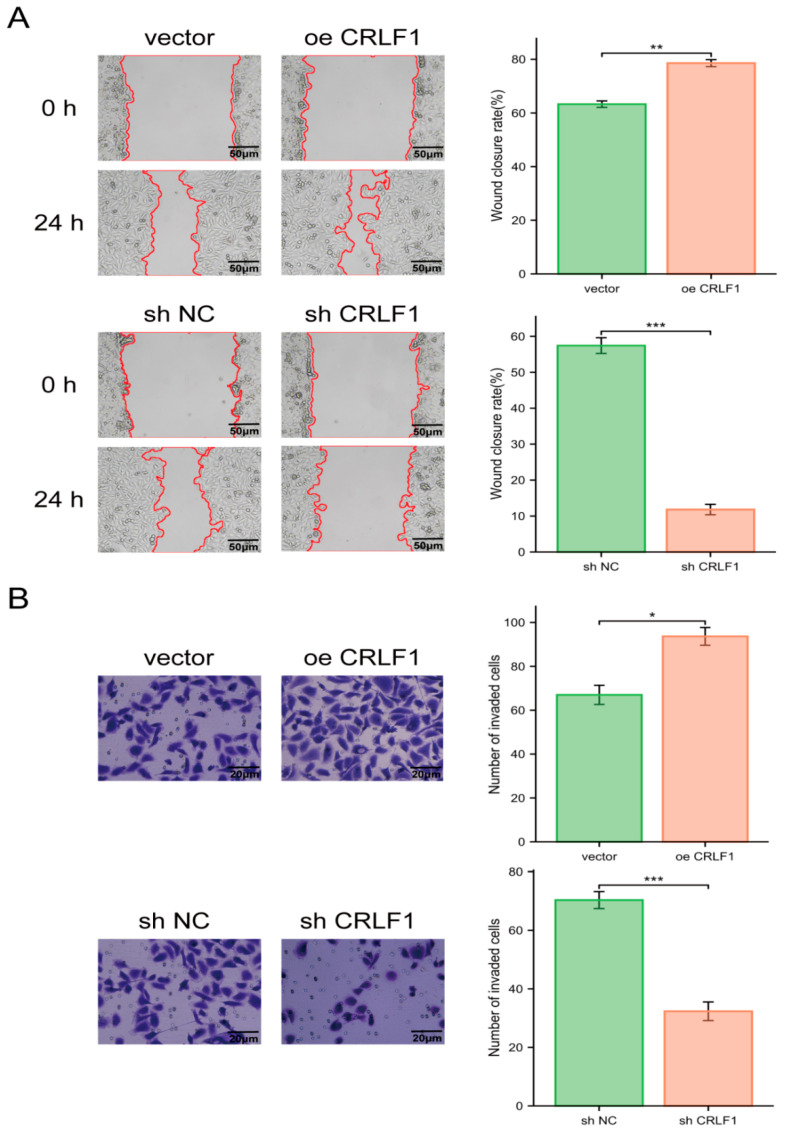
CRLF1 triggers the migration and invasion of PCa cells. (**A**) Wound healing assays illustrated that CRLF1 upregulation in DU145 cells promoted migration, whereas knockdown of CRLF1 inhibited migration (*n* = 3). The red lines indicate the wound edges used for migration assessment. (**B**) Transwell assays showed that CRLF1 upregulation significantly increased invasion, while CRLF1 knockdown reduced invasive capacity (*n* = 3). Data are represented as mean ± SD. * *p* < 0.05; ** *p* < 0.01; *** *p* < 0.001.

In wound healing assays, CRLF1 overexpression improved the migration capability of PCa cells, while a significant decrease in CRLF1 expression impaired this capacity (Figure 3A). Similarly, Transwell assays demonstrated that CRLF1 overexpression noticeably elevated the invasive capability of PCa cells, whereas CRLF1 suppression inhibited cell invasion (Figure 3B).

### 3.3. CRLF1 Is Positively Associated with COMP Expression in PCa Cells

**Figure 4:**
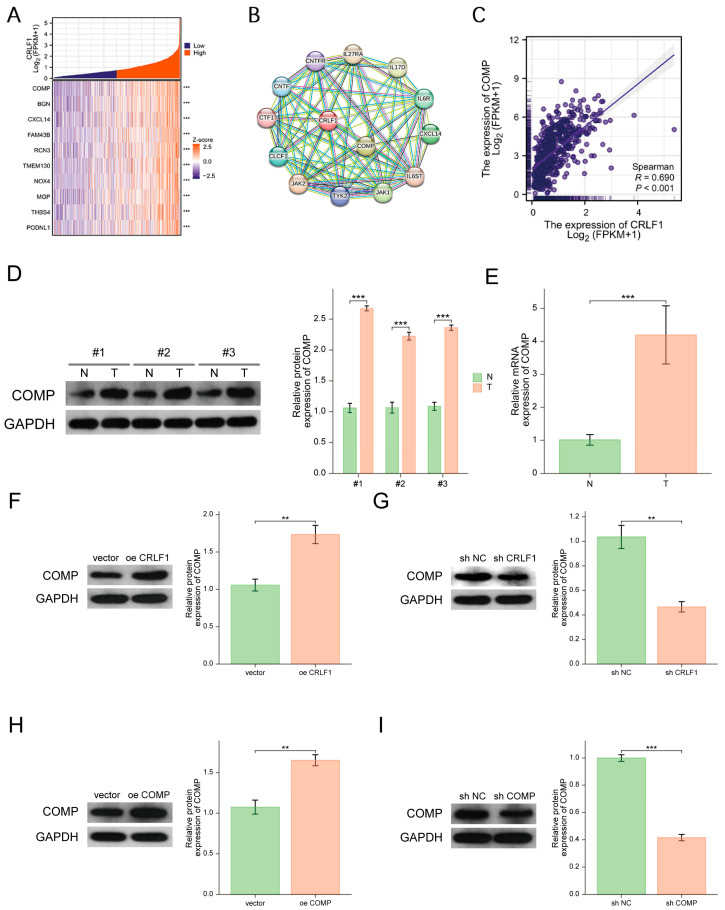
Positive association between CRLF1 and COMP in PCa cells. (**A**) Heatmap of the 10 genes most positively correlated with CRLF1 in PCa. (**B**) STRING protein–protein interaction analysis identifying an interaction between CRLF1 and COMP. (**C**) Positive correlation between CRLF1 and COMP expression (Spearman r = 0.690). (**D**,**E**) WB (*n* = 3) and qRT-PCR (*n* = 17) of COMP in PCa tissues and neighboring healthy tissues. (**F**,**G**) WB analysis of COMP level in DU145 cells following CRLF1 overexpression or knockdown (*n* = 3). (**H**,**I**) WB of COMP overexpression and suppression in DU145 cells (*n* = 3). Data are represented as mean ± SD. ** *p* <0.01; *** *p* <0.001. The corresponding uncropped original Western blot images with molecular weight markers are provided in Supplementary Figure S2I–S.

To gain a deeper comprehension of the possible biological activities of CRLF1 in PCa, we conducted co-expression trials using the TCGA-PRAD dataset. We created heatmaps to visualize the top 10 genes significantly positively correlated with CRLF1 in PCa (Figure 4A). To further clarify the biological processes related to CRLF1, we created a protein–protein interaction (PPI) network focusing on CRLF1 in PCa using the STRING database. Notably, the analysis illustrated a potential association between CRLF1 and COMP (Figure 4B). By detecting the levels of CRLF1 and COMP in the TCGA-PRAD dataset, we determined a significant positive link between the two in PCa (Figure 4C). WB and qRT-PCR analysis confirmed that the COMP level was significantly heightened in PCa tissues compared to healthy tissues (Figure 4D,E). Additionally, in DU145 cells overexpressing CRLF1, COMP expression was significantly upregulated (Figure 4F), further supporting the positive association between CRLF1 and COMP. Conversely, reduced CRLF1 expression in DU145 cells was associated with COMP inhibition (Figure 4G). Subsequently, we established stable DU145 cell lines with upregulated or downregulated COMP expression and validated the gene editing efficiency using WB (Figure 4H,I).

### 3.4. CRLF1 Promotes Cell Growth and Invasion via Its Interaction with COMP in PCa Cells

**Figure 5:**
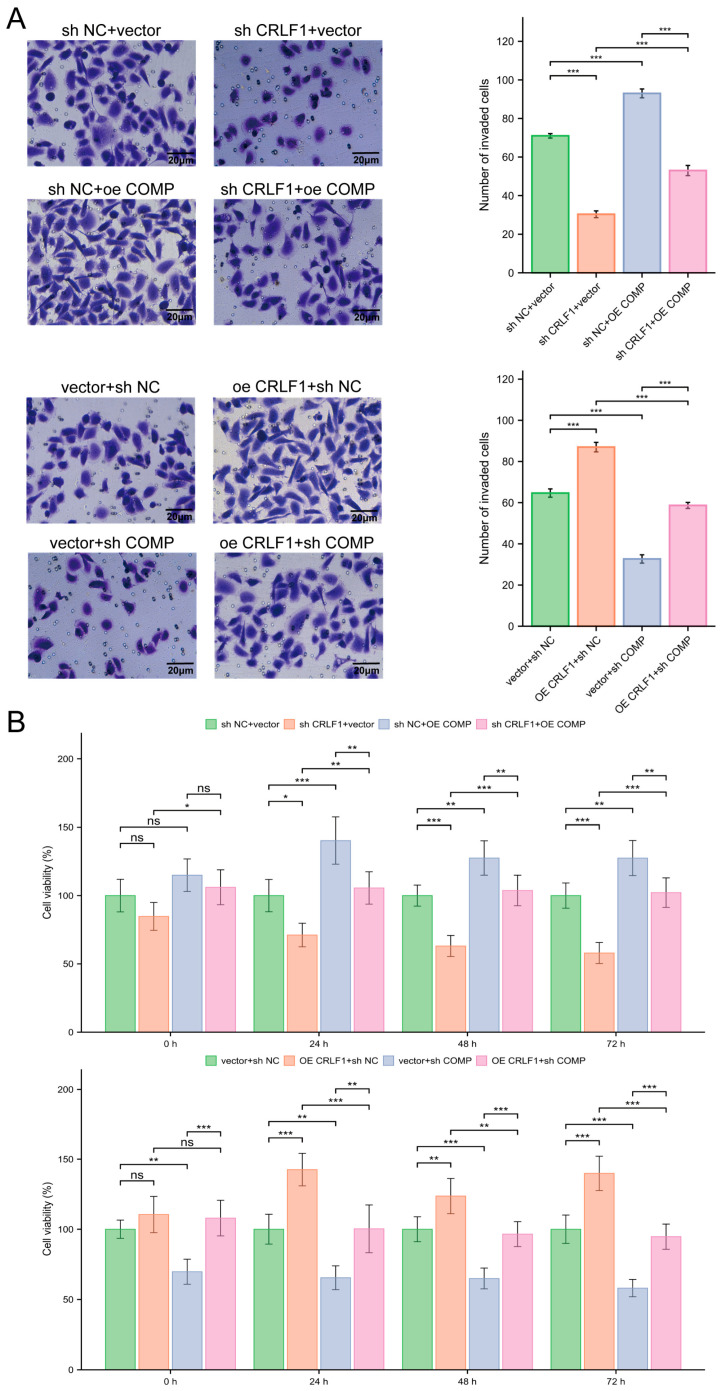
CRLF1 promotes invasion and cell proliferation in PCa cells by interacting with COMP. (**A**) After knockdown or overexpression of CRLF1 in DU145 cells, reciprocal upregulation or downregulation of COMP was performed. The invasive capacity of transfected DU145 cells was assessed using Transwell assays (*n* = 3). (**B**) Cell proliferation of transfected DU145 cells was measured by CCK-8 assay at 0, 24, 48 and 72 h (*n* = 5). Values were normalized to the corresponding control group at each time point and expressed as percentage viability. Data are reported as mean ± SD. * *p* < 0.05; ** *p* < 0.01; *** *p* <0.001; ns, no statistical difference.

Transwell assays confirmed that the enhanced invasive capability induced by CRLF1 overexpression could be reversed when COMP was silenced, whereas the attenuated invasion observed upon CRLF1 knockdown was restored by COMP overexpression (Figure 5A). Further investigations using CCK-8 assays revealed that the proliferative enhancement triggered by elevated CRLF1 levels was blocked after COMP silencing, while COMP overexpression reversed the proliferative defect caused by reduced CRLF1 levels (Figure 5B).

### 3.5. This Study Utilizes the Functional Enrichment Analysis and TCGA-PRAD Dataset

**Figure 6:**
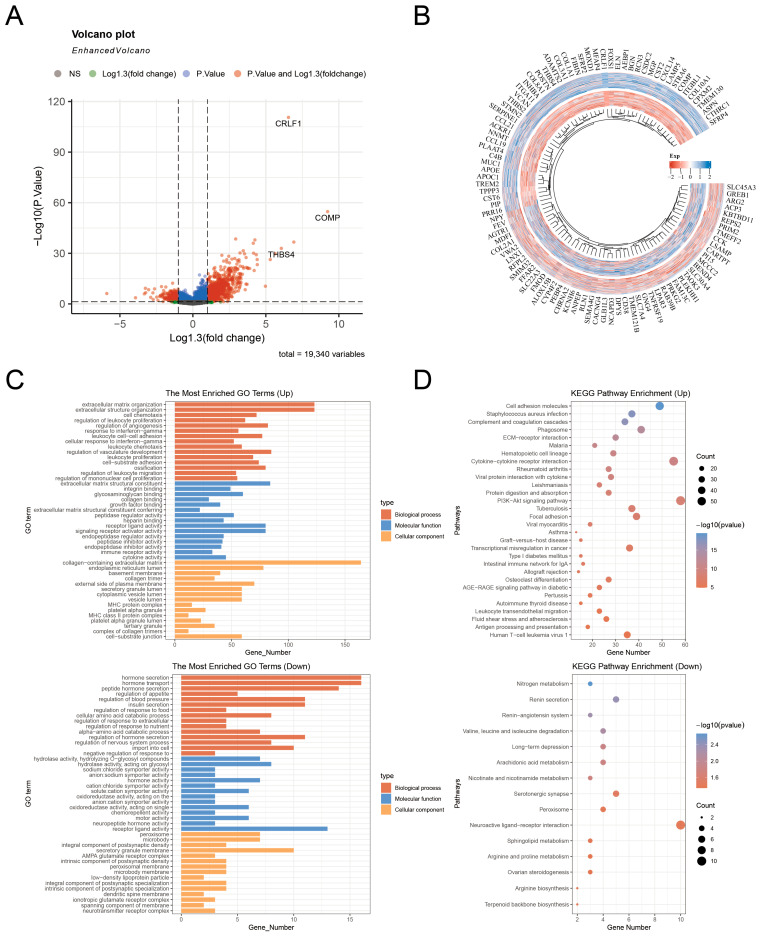
Identification of CRLF1-related genes and functional enrichment analysis based on the TCGA-PRAD dataset. (**A**) A volcano plot of the TCGA-PRAD dataset showing genes associated with CRLF1 expression. (**B**) Circular heatmap illustrating CRLF1-related genes. (**C**,**D**) KEGG and GO analyses of genes positively linked to CRLF1 in PCa. (**E**,**F**) KEGG and GO analyses of genes negatively linked to CRLF1 in PCa. GO comprises cellular component (CC), biological process (BP), and molecular function (MF).

The TCGA-PRAD dataset is employed to determine genes that illustrate a strong link with CRLF1 in PCa, with a specific focus on genes that show significant upregulation or downregulation, where the log2|FC| value is greater than 1.3. These genes are subsequently visualized using volcano plots and circular heatmaps (Figure 6A,B). A thorough investigation is performed by applying KEGG and GO analyses to these genes. The GO analysis reveals that the overexpressed genes are primarily enriched in biological processes related to the extracellular matrix with collagen (Figure 6C). The KEGG analysis indicates that the upregulated genes are significantly enriched in pathways predominantly correlated with the PI3K/AKT pathway and adhesion complexes (Figure 6D). The GO analysis shows that the suppressed genes are mainly enriched in processes related to hormonal regulation in the body and the transport of substances (Figure 6E). KEGG further analysis highlights the significant enrichment of suppressed genes, which are primarily involved in amino acid and lipid metabolism (Figure 6F).

### 3.6. CRLF1 Activates the FAK/PI3K/AKT Signaling Pathway in PCa Cells via COMP

**Figure 7:**
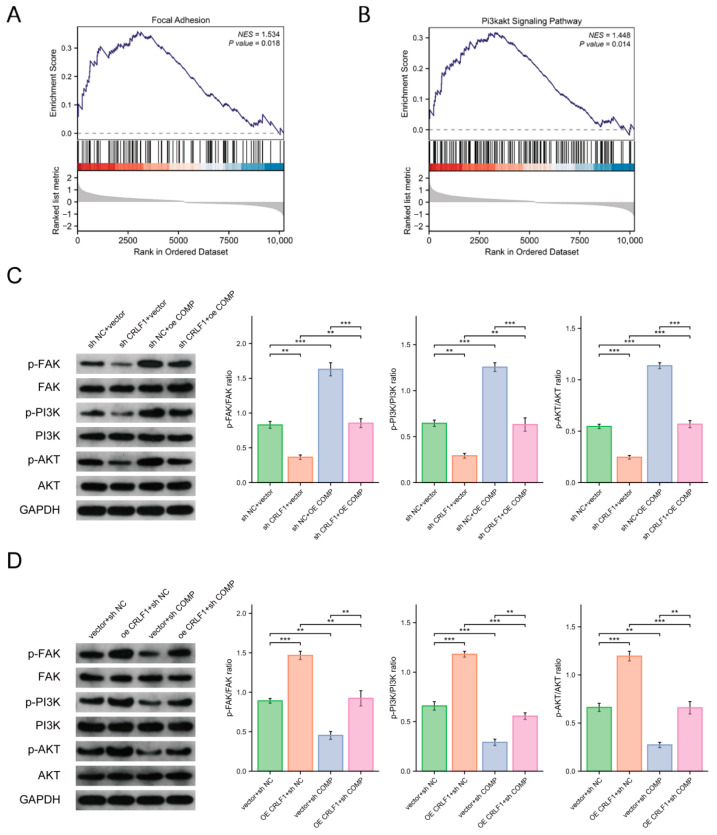
CRLF1 activates the FAK/PI3K/AKT pathway in PCa cells through COMP. (**A**,**B**) GSEA illustrated that CRLF1 expression was significantly related to focal adhesion formation and PI3K/AKT pathway stimulation. (**C**,**D**) In DU145 cells, COMP was overexpressed or silenced following CRLF1 knockdown or overexpression, respectively, and protein levels of p-PI3K, PI3K, p-AKT, FAK, p-FAK, and AKT were re-evaluated by WB. Data are represented as mean ± SD. ** *p* < 0.01, *** *p* < 0.001. The corresponding uncropped original Western blot images with molecular weight markers are provided in Supplementary Figure S2T,U and Supplementary Figure S3A–L.

The focal adhesion and PI3K/AKT pathways were significantly enriched in PCa samples with overexpressed CRLF1, according to GSEA (Figure 7A,B). WB showed that neither overexpression nor silencing of CRLF1 altered the total protein levels of FAK, PI3K, and AKT. However, the increased phosphorylation resulting from CRLF1 overexpression was inhibited by COMP knockdown (Figure 7C). Conversely, overexpression of COMP restored the reduced phosphorylation levels caused by CRLF1 downregulation (Figure 7D). These findings suggest that CRLF1 promotes prostate cancer progression in association with COMP and activation of the FAK/PI3K/AKT pathway.

### 3.7. CRLF1 Enhances the In Vivo Tumor Growth of PCa by Stimulating the FAK/PI3K/AKT Pathway via COMP

**Figure 8:**
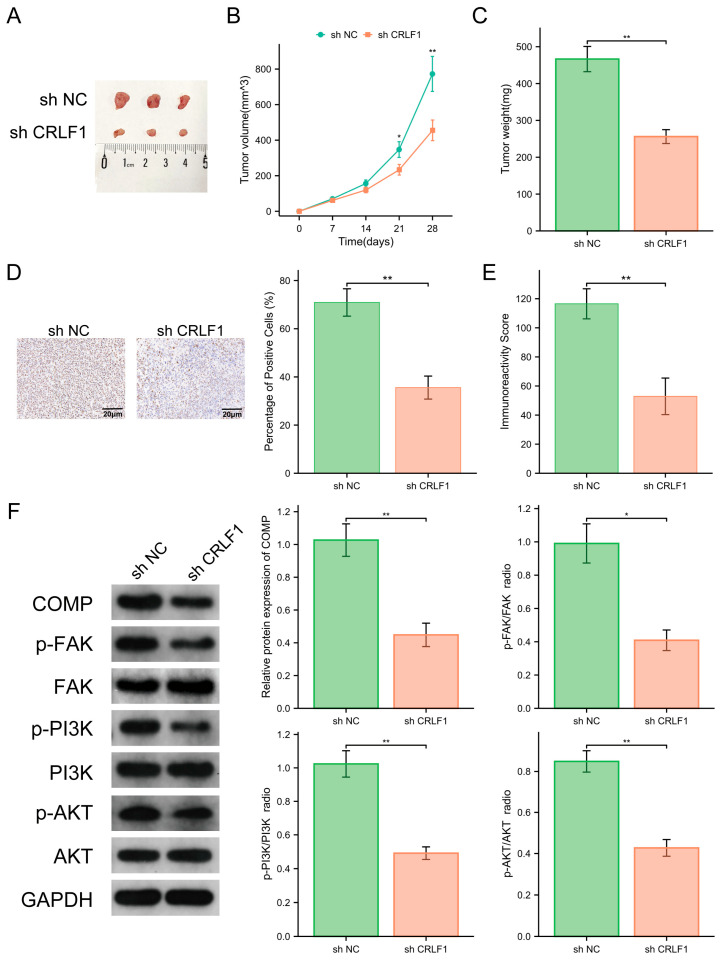
CRLF1 triggers tumor growth and activates the FAK/PI3K/AKT pathway in vivo in PCa. (**A**) Subcutaneous xenograft models were generated using DU145 cells transfected with shNC or shCRLF1. (**B**,**C**) Tumor volume and weight were measured in both groups, with three mice per group. (**D**,**E**) Immunohistochemical staining of Ki67 in xenograft tissues illustrated that the positive rate of cells and the immunoreactivity score in the gene knockout group were significantly lowered compared to the control. (**F**) WB assessed the protein levels of COMP, p-FAK, PI3K, p-AKT, FAK, p-PI3K, and AKT in xenograft tissues. Data are represented as mean ± SD. * *p* <0.05, ** *p* <0.01. The corresponding uncropped original Western blot images with molecular weight markers are provided in Supplementary Figure S3M–U.

To estimate the influence of CRLF1 on PCa progression in vivo, a heterotransplantation model was created for experimental analysis. The findings revealed that CRLF1 knockdown markedly suppressed the growth rate, weight, and volume of the heterotransplant tumors (Figure 8A–C). In addition, we performed immunohistochemical staining for Ki67 using subcutaneous tumors, and the outcomes illustrated that the positive rate of Ki67 cells and the immunoreactivity score of the knockdown group were significantly lowered compared to the control group (Figure 8D,E). Subsequently, protein extracts from subcutaneous tumor tissues were subjected to WB to estimate the expression and phosphorylation status of COMP, FAK, PI3K, and AKT in PCa heterotransplant tissues. The results indicated that CRLF1 silencing had no significant influence on the total protein levels. However, CRLF1 knockdown caused a decrease in COMP expression and significantly decreased the phosphorylation levels of PI3K, FAK, and AKT (Figure 8F).

## 4. Discussion

PCa is a predominant malignancy among men globally, with an estimated occurrence of about 1.4 million new cases detected yearly, making it a significant public health concern [24,25]. Despite progress in elucidating the molecular mechanisms of PCa, there is still an urgent requirement for innovative therapeutic strategies capable of successfully targeting the key signaling pathways that drive tumor progression and participate in treatment resistance [26,27,28].

In this investigation, we explored the function of CRLF1 in PCa, demonstrating its substantial contribution to enhancing tumor proliferation and metastatic potential via modulation of COMP and the consequent stimulation of the FAK/PI3K/AKT signaling cascade. These outcomes offer fresh perspectives on the molecular mechanisms underlying CRLF1 in PCa development and underscore its viability as a marker for both diagnosis and prognosis.

First, our outcomes reveal a significant elevation in CRLF1 expression levels within PCa tissues relative to normal controls, as validated through WB and qRT-PCR assays. This overexpression correlates with enhanced cellular proliferation, invasion, and reduced apoptosis in vitro, supported by Transwell invasion, wound healing, CCK-8 assays and flow cytometric apoptosis detection. The in vivo xenograft model further substantiated these results, showing that inhibition of CRLF1 effectively suppresses tumor progression. These observations align with the known involvement of CRLF1 in cancer progression and treatment resistance in other contexts, though its specific role in PCa has been underexplored. Our study thus establishes CRLF1 as a key promoter of PCa aggressiveness. In addition, qRT-PCR validation in clinical prostate cancer specimens demonstrated that CRLF1 mRNA was significantly elevated, suggesting that CRLF1 upregulation occurs, at least in part, at the mRNA level. Notably, although TCGA-based pan-cancer analysis showed increased CRLF1 expression in several tumor types, decreased expression was observed in some cancers, which may reflect tissue-specific biological characteristics, tumor heterogeneity, and differences in the tumor microenvironment. The additional public-dataset analyses indicated that CLCF1-related findings may vary across cohorts, suggesting that the contribution of the canonical CLCF1-associated signaling context in prostate cancer requires further investigation. This interpretation is consistent with the established biology of the CRLF1–CLCF1 signaling framework [29] and with prior reports suggesting context-dependent roles of CLCF1 across different tumor types [30,31,32]. In contrast, the positive association between CRLF1 and COMP was reproduced in an external dataset, further supporting the relevance of the CRLF1/COMP-associated axis in our study (Supplementary Figure S1).

Our results highlight a positive association between CRLF1 and COMP in prostate cancer. Analyzing the protein interaction networks and the TCGA-PRAD dataset illustrated a robust positive link between CRLF1 and COMP expression levels. Functional experiments revealed that COMP inhibition reverses the pro-tumorigenic effects induced by CRLF1 overexpression, including increased cell growth and invasion. This suggests that CRLF1 exerts its oncogenic functions, at least in part, by modulating COMP levels. COMP, functioning as an extracellular matrix protein, potentially mediates interactions within the tumor microenvironment; however, its specific involvement in PCa signaling pathways necessitates additional investigation [33,34].

The FAK/PI3K/AKT cascade is a well-characterized molecular axis that governs key oncogenic functions (e.g., cell survival, growth, motility, invasiveness, and EMT control) [35,36,37]. In the present investigation, we discovered a notable association between CRLF1 overexpression and the stimulation of adherens junctions as well as the PI3K/AKT signaling cascade, utilizing extensive bioinformatic methodologies, such as GO, KEGG, and GSEA. Guided by these observations, we conducted in vitro and in vivo assessments to assess protein expression within the FAK/PI3K/AKT signaling network. Our findings indicate that CRLF1 overexpression in PCa cells stimulates FAK/PI3K/AKT signaling, whereas CRLF1 knockdown attenuates its activity. Mechanistically, we suggest that CRLF1 facilitates PCa progression via the FAK/PI3K/AKT axis. More specifically, the evidence implies that CRLF1-induced upregulation of COMP contributes to pathway activation, thereby reinforcing tumorigenic and invasive behaviors in PCa cells. These outcomes align with the established activity of the FAK/PI3K/AKT pathway in transducing extracellular cues to PCa advancement. Collectively, these outcomes illustrate that CRLF1 participates in the development of invasive phenotypes in PCa by triggering the stimulation of the FAK/PI3K/AKT pathway. However, the precise molecular intermediates linking CRLF1, COMP, and FAK/PI3K/AKT activation require further elucidation. Future studies should aim to dissect these specific interactions, for example, by examining detailed phosphorylation events or employing pathway-specific inhibitors.

The clinical implications of our work are substantial. CRLF1’s association with poor prognostic features, such as increased invasion and tumor growth, sets it as a prospective biomarker for PCa diagnosis and risk stratification. The correlation between CRLF1 and COMP expression in patient datasets further supports their combined use in prognostic models. Additionally, targeting the CRLF1/COMP/FAK/PI3K/AKT axis may offer new therapeutic avenues for advanced or resistant PCa. For instance, small molecule inhibitors or antibodies against CRLF1 or COMP could be explored in preclinical settings.

From a translational perspective, our findings suggest that CRLF1 may serve as a potential biomarker for PCa progression and provide a rationale for future studies exploring whether CRLF1, COMP, or related downstream signaling components may represent actionable therapeutic targets. Although our data do not establish an immediate therapeutic application, they support further investigation of the CRLF1/COMP-associated signaling context in prostate cancer.

Nevertheless, this investigation has limitations. External validation of clinicopathological and survival associations was limited by the lack of sufficiently complete and consistently matched clinical annotations in the available public cohorts. The experimental models used, while informative, may not completely recapitulate the complex heterogeneity of human PCa. In addition, only the prostate cancer cell lines currently available in our laboratory were included in this study. Although DU145 was selected as the principal model because it showed the highest endogenous CRLF1 expression in our screening, validation in additional models, such as LNCaP and C4-2, would further strengthen the generalizability of our findings. Similarly, although CRLF1 expression was also elevated in 22RV1 and PC-3 cells, they were not included in downstream mechanistic validation; 22RV1 harbors an AR-variant background and PC-3 is PTEN-deficient, factors which may complicate the interpretation of CRLF1-associated signaling. Moreover, the functional assays were primarily conducted in cell lines, and the in vivo model relied on xenografts, which might not mirror the tumor microenvironment in patients. Although CRLF1 mRNA was confirmed to be elevated in clinical prostate cancer specimens, the precise mechanism underlying its upregulation was not investigated beyond the mRNA level, and whether altered translation efficiency or enhanced protein stability also contributes remains unclear. In addition, post-translational modification signatures of CRLF1 were not analyzed in the present study. In our experimental system, COMP appears to function as a mediator associated with CRLF1 oncogenic signaling, although the precise molecular mechanism remains to be clarified. Additionally, exploring the upstream regulators of CRLF1 in PCa and determining whether CRLF1 regulates FAK/PI3K/AKT at the mRNA level remain important areas for future study.

## 5. Conclusions

In summary, our results indicate a marked elevation in CRLF1 expression within PCa tissues, correlating with unfavorable clinical outcomes. Utilizing in vitro and in vivo models, we have established that CRLF1 contributes to tumor progression in association with COMP and activation of the FAK/PI3K/AKT pathway. The suppression of CRLF1 or its downstream effector COMP significantly inhibits cancer cell proliferation, invasion, and tumor formation. Consequently, our findings support the relevance of a CRLF1/COMP-associated mechanism in prostate cancer progression and suggest that CRLF1 may represent a potential biomarker and therapeutic target in PCa.

## Figures and Tables

**Table 1:** Primer sequences.

Gene	GenBank ID	Primer (5′→3′)	Product (bp)	Application
CRLF1	NM_004750	F: CTCTCCCGTGTACTCAACGC	144	qRT-PCR
		R: GGGCAGGCCAACATAGAGG		
COMP	NM_000095	F: GATCACGTTCCTGAAAAACACG	148	qRT-PCR
		R: GCTCTCCGTCTGGATGCAG		
GAPDH	NM_014364	F: TGTGGGCATCAATGGATTTGG	116	qRT-PCR (Reference)
		R: ACACCATGTATTCCGGGTCAAT		

## Data Availability

The data supporting the findings of this study are included in the article and its Supplementary Materials. Additional data are available from the corresponding author upon reasonable request.

## Supplementary Materials

The following supporting information can be downloaded at: https://www.mdpi.com/article/10.3390/cancers18091395/s1, Figure S1: External validation of CRLF1, COMP, and CLCF1 expression patterns and correlation analyses in prostate cancer datasets. (A) CRLF1 expression in normal and prostate cancer tissues in the independent GEO cohort GSE46602. (B) COMP expression in normal and prostate cancer tissues in GSE46602. (C) CLCF1 expression in normal and prostate cancer tissues in GSE46602. (D) Correlation analysis between CRLF1 and COMP in GSE46602. (E) Correlation analysis between CRLF1 and CLCF1 in GSE46602. (F) COMP expression in normal and prostate cancer tissues in the TCGA-PRAD cohort. (G) CLCF1 expression in normal and prostate cancer tissues in the TCGA-PRAD cohort. (H) Correlation analysis between CRLF1 and CLCF1 in the TCGA-PRAD cohort. Expression values are presented as log2-transformed normalized expression values. Correlations were assessed using Spearman’s rank correlation coefficient. * *p* < 0.05, ** *p* < 0.01, *** *p* < 0.00; ns, no statistical difference. Figures S2 and S3: The corresponding uncropped original Western blot images with molecular weight markers.

## Abbreviations

ANOVA	Analysis of variance.
AKT	Protein kinase B.
CCK-8	Cell Counting Kit-8.
COMP	Cartilage Oligomeric Matrix Protein.
CRLF1	Cytokine-like receptor family 1.
DEGs	Differentially expressed genes.
GEO	Gene Expression Omnibus.
GO	Gene Ontology.
GSEA	Gene set enrichment analysis.
IHC	Immunohistochemistry.
KEGG	Kyoto Encyclopedia of Genes and Genomes.
mTOR	Mechanistic target of rapamycin.
PCa	Prostate cancer.
PFI	Progression-Free Interval.
PI3K	Phosphoinositide 3-kinase.
PPI	Protein–protein interaction.
PSA	Prostate-specific antigen.
PVDF	Polyvinylidene difluoride.
qRT-PCR	Quantitative real-time polymerase chain reaction.
SD	Standard deviation.
TCGA	The Cancer Genome Atlas.
WB	Western blot.
