# Supplementary material for: CRLF1 Drives Prostate Cancer Progression via COMP-Mediated Activation of the FAK/PI3K/AKT Signaling Pathway
Source: Cancers (Basel). 2026 Apr 28;18(9):1395. doi: 10.3390/cancers18091395 (PMC13162994; doi:10.3390/cancers18091395)
Supplement: Supplementary file 1 [file cancers-18-01395-s001.zip › Supplementary Figure S1.pdf]

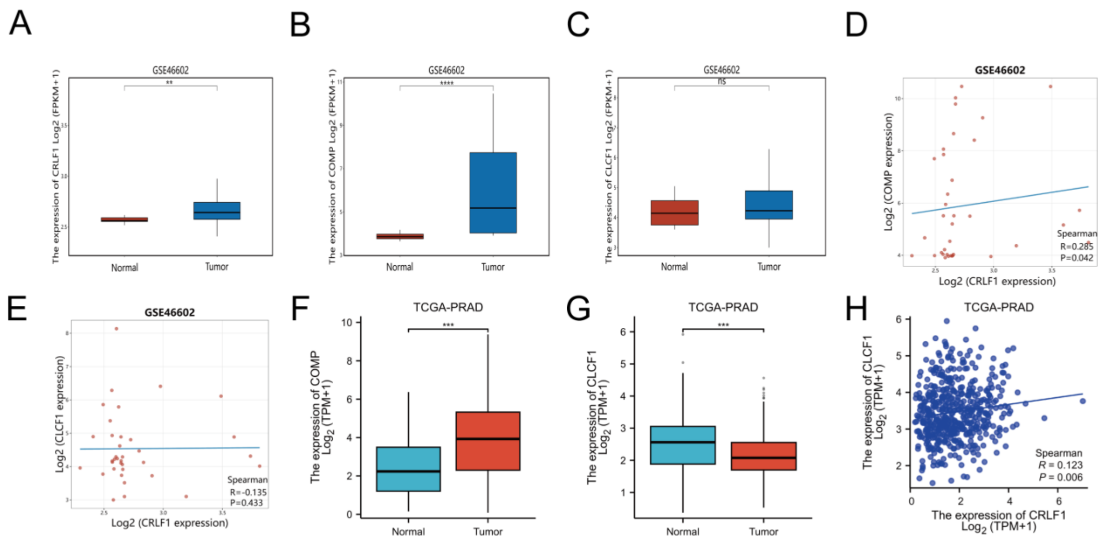

Supplementary Figure S1. External validation of CRLF1, COMP, and CLCF1 expression patterns and correlation analyses in prostate cancer datasets. (A) CRLF1 expression in normal and prostate cancer tissues in the independent GEO cohort GSE46602. (B) COMP expression in normal and prostate cancer tissues in GSE46602. (C) CLCF1 expression in normal and prostate cancer tissues in GSE46602. (D) Correlation analysis between CRLF1 and COMP in GSE46602. (E) Correlation analysis between CRLF1 and CLCF1 in GSE46602. (F) COMP expression in normal and prostate cancer tissues in the TCGA-PRAD cohort. (G) CLCF1 expression in normal and prostate cancer tissues in the TCGA-PRAD cohort. (H) Correlation analysis between CRLF1 and CLCF1 in the TCGA-PRAD cohort. Expression values are presented as log2-transformed normalized expression values. Correlations were assessed using Spearman's rank correlation coefficient. *p <0.05, ** p <0.01, ***p < 0.00; ns, no statistical difference.
